# Polymorphisms of Aldose Reductase (ALR2) Regulatory Gene are Risk Factors for Diabetic Retinopathy in Type-2 Diabetes Mellitus Patients in Bali, Indonesia

**DOI:** 10.2174/1874364101812010281

**Published:** 2018-10-18

**Authors:** Desak Made Wihandani, Ketut Suastika, I Nyoman Agus Bagiada, Safarina G. Malik

**Affiliations:** 1 Department of Biochemistry, Faculty of Medicine, Udayana University, Jl. PB Sudirman, Denpasar 80232, Bali, Indonesia; 2 Division of Endocrinology and Metabolism, Department of Internal Medicine, Faculty of Medicine, Udayana University, Jl. PB Sudirman, and Sanglah Hospital, Jl. Kesehatan, Denpasar, Bali, Indonesia; 3 Eijkman Institute for Molecular Biology, Jl. Diponegoro 69, Jakarta 10430, Indonesia

**Keywords:** ALR2 gene, C(-104)T polymorphism, Diabetic retinopathy, Type-2 diabetes mellitus, C(-9)G polymorphism, PCR

## Abstract

**Aim:**

The study aimed to elucidate whether the polymorphisms of the aldose reductase regulatory gene were risk factors for Diabetic Retinopathy (DR) in type-2 diabetes mellitus (T2DM) patients in Bali.

**Methods:**

This is a case-control study including 35 cases of T2DM patients with DR paired with 35 cases with non-DR as controls. PCR analysis and DNA-sequencing were carried out to detect the C(-106)T and C(-12)G polymorphisms at the regulatory region of Aldose Reductase (ALR2) gene. Genotype and allele distributions were analyzed by Chi-squared test and independent t-and Mann-Whitney U tests were used to analyze other data.

**Results:**

Among all subjects in both groups, the baseline characteristics were homogenous except for systolic blood pressure, fasting blood glucose and 2-hours post-prandial blood glucose. This study found two polymorphisms, C(-104)T and C(-9)G, in the regulatory region of ALR2 gene. The result showed that the C(-104)T polymorphism was a risk factor for DR (OR=36; 95% CI = 4.43-292.85; *p*=0.001), but not the C(-9)G polymorphism (OR=1.28; 95% CI=0.48-3.38; *p*=0.621). Other findings in the study revealed that CC/CC haplotype is a protective factor for DR (OR=0.198; *p*=0.002), whereas CT/CC and CT/CG haplotypes as risk factors for DR with OR=15.58; *p*=0.002 and OR=2.29; *p*=0.005 respectively.

**Conclusion:**

It can be concluded that C(-104)T polymorphism in the regulatory region of Aldose Reductase (ALR2) gene was the risk factor for DR among T2DM patients in Bali, Indonesia. However, small sample size, systolic blood pressure, fasting blood glucose and 2-hours post-prandial blood glucose could affect our finding.

## 1 INTRODUCTION

The incidence of diabetes mellitus, especially Type-2 Diabetes Mellitus (T2DM) increases not only in developed countries but also in developing countries. Indonesia as one of a developing country has been estimated to be the fourth country with the highest number of diabetic cases in 2030. Various efforts have been attempted to overcome this problem, including new drugs development as well as changes in dietary pattern and lifestyle, but the number of cases is still increasing [1].

Type-2 DM is a multifactorial disease with micro and macro-vascular complications. The commonest microvascular complication in DM is Diabetic Retinopathy (DR) and the leading cause of blindness among diabetic patient in the productive age. DR can occur in the early stage of the disease in diabetic patients with well-controlled blood sugar level, while other patients who have diabetes for many years, do not complicate with DR. This suggests that the occurrence of DR cannot be explained based on the hyperglycaemic state alone. Several evidences have shown that complications of DM are also affected by genetic factors [2, 3].

Aldose Reductase (ALR2) is a rate-limiting enzyme in the polyol pathway. This enzyme catalyzes the reduction of carbonyl group into their alcohol product, converts D-glucose into sorbitol and galactose into galactycol [4, 5]. Many studies of genetic polymorphism showed that ALR2 is the risk factor for DR and other microvascular complications. ALR2 gene polymorphisms at regulatory-region have been used as a marker for genetic susceptibility of DM complications. However, several studies reported inconsistent results on the correlation between DM complications and ALR2 polymorphisms, probably contributed by ethnics differences [6-9]. Common polymorphisms of ALR2 gene in its regulatory region have been reported associated with microvascular complications in DM, with several studies reported to correlate with DR.

Aldose Reductase (ALR2) gene polymorphism (C→T) at position -106 of the promoter region has been reported previously [10]. The frequency of the genotypes was found to be significantly higher in diabetic patients with DR than among those without DR. Another study has also reported that C(-106)T and C(-12)G polymorphisms were found in the Chinese population, although analysis of these two polymorphisms as a haplotype was not carried out [2]. Identification of genetic risk factors of DR will help in understanding the complex complication of type-2 DM.

This study was conducted based on the polymorphic characteristics of ALR2 gene in the regulatory region. Currently, studies on ALR2 polymorphisms, expression, and haplotype identification associated with microvascular complications of T2DM in Indonesia are still limited.Therefore, the primary objective of this study was to determine whether polymorphisms at the regulatory region of ALR2 gene were risk factors for DR in T2DM patients in Bali, Indonesia.

## 2 MATERIAL AND METHODS

### 2.1 Study Subjects and Measurements

A total of 70 subjects (35 T2DM patients with DR and 35 T2DM without DR) with Balinese ethnic were enrolled from Sanglah General Hospital, Public Health Care Center at the West and East Denpasar between February 2013 and February 2014. The sample size was calculated using the formula for case study control for hypothesis test against Odds Ratio [11]. The inclusion criteria for T2DM patients in this study in accordance with American Diabetes Association (ADA), 2011: Balinese ethnic with age between 35-65 years old and have been diagnosed with T2DM for 5-15 years [12].T2DM patients with stroke and myocardial infarction were excluded from the study. The ophthalmologist diagnosed diabetic retinopathy based on American Academy of Ophthalmology (AAO), 2010 criterion [13].

Body Mass Index (BMI) was calculated as body weight in kilograms divided by the square of height in meters. Body weight was measured using a digital scale, while body height was assessed using stature meter. Waist Circumference (WC) was measured using flexible non-elastic tape precisely at the middle level of the abdomen. Systolic and diastolic blood pressures were measured in a sitting position for two times using a mercury sphygmomanometer.

Fasting and 2 hours after meal blood samples were collected and separated into two vacutainer tubes with EDTA. One tube was used to examine blood glucose level and HbA1C, while the other was stored at -20^o^C for DNA isolation and Polymerase Chain Reaction (PCR) analysis. The study protocol has been approved by the Ethics Commission of the Faculty of Medicine, Udayana University/Sanglah General Hospital (No. 759/UN.14.2/Litbang/2012), and all subjects were provided with written informed consent.

### 2.2 Study Design

A case-control study was designed, to elucidate whether the ALR2 polymorphisms were risk factors for DR in T2DM patients in Bali, Indonesia.

### 2.3 Study Protocol

PCR analysis followed by DNA sequencing was used to detect polymorphisms. Genotyping of C(-106)T polymorphism of the ALR2 gene was performed using primer pair 5’-TTC GCT TTC CCA CCA GAT-3’ between nucleotide -225 and -235 and 5’CGC CGT TGT TGA GCA CGA GAC-3’ between nucleotide 50 and 71 that amplify a 326 base pair fragment. The PCR conditions consisted of pre-denaturation at 95^o^ C for 2 minutes, followed by 35 cycles of denaturation at 95^o^C for 60 seconds, annealing at 53^o^C for 60 seconds, and extension at 72^o^C for 60 seconds. The final extension was at 72^o^C for 7 minutes [10].

Genotyping of ALR2 C(-12)G polymorphism was performed using primer pair 5'-CGC TAA AGC TTT CGC TTT CCC ACC AGA TAC AGC-3' and 5'-ATG GCT GCA GCG CTC CCC AGA CCC CCG CCC AGT-3'. The PCR conditions were as follows: denaturation at 95^o^ C for 2 minutes, followed by 35 cycles of denaturation at 95 ^o^ C for 60 seconds, annealing at 55^o^C for 60 seconds, and a final extension at 72^o^C for 60 seconds. The final extension was at 72^o^C for 1 minute [2].

All PCR products were then purified and sequenced. Polymorphisms were analyzed based on the electropherograms, confirmed by BLAST with the reference sequence in gene bank (sequence ID: gb|U72619.1|HSU72619) as reported by Ko *et al*., 1995 [14].

### 2.4 Statistical Analysis

Kolmogorov–Smirnov Test was used to evaluate the normality of variables and normally distributed values were expressed as a mean ± standard deviation. Genotype and allele distributions were analyzed using Chi-square test, while other data were analyzed using independent t- and Mann-Whitney U tests with *p*<0.05 was considered as significantly different. Fisher’s exact test was applied to determine whether or not the genotype distribution has departed from the Hardy-Weinberg equilibrium.

## 3 RESULTS

### 3.1 Subject Characteristics

Thirty-five subjects with DR (cases) and 35 subjects without DR (control) were chosen randomly in this study. As shown in Table **1**, baseline characteristics of the subjects between DR and non-DR group based on sex, family history of DM, age, duration of DM, waist circumference, BMI and HbA1c level showed no significant differences as analyzed using independent t-, Mann-Whitney U and Chi-square tests (*p*>0.05). However, systolic blood pressure, Fasting Blood Glucose (FBG) and 2-Hours post-prandial Blood Glucose (2hppBG) were significantly higher in the case group as compared to control group (*p*<0.05).

### 3.2 Polymorphisms in ALR2 Gene at the Regulatory Region

The genotyping result of C(-106)T and C(-12)G polymorphisms at the regulatory region of ALR2 gene was determined by DNA sequencing as shown in Fig. (**1**). We found two polymorphisms, C(-104)T and C(-9)G at the regulatory region of ALR2 gene based on the electropherogram results, which were confirmed by BLAST. Further analysis using multiple alignments found that these two polymorphisms were identical with the previously reported C(-106)T rs 759853 and C(-11)G. Additionally, this study found 25 (26%) CT heterozygous, 70 (72%) CC homozygous and 2 (2%) C deletion without any TT homozygous at position -104. Thirty-two (33%) CG heterozygous, 62 (64%) CC homozygous and 3 (3%) GG homozygous were found at position -9. Complete results of ALR2 polymorphisms are presented in Table **2**.

Based on genotypic distribution analysis, both C(-104)T and C(-9)G polymorphisms showed no significant departure from Hardy-Weinberg Equilibrium (HWE) with *p*=0.13 and 0.64 respectively.

### 3.3 Risk Factors for Diabetic Retinopathy

Chi-square test was used to determine the role of C(-104)T and C(-9)G polymorphisms as risk factors for DR in T2DM patients. Table **3** shows that C(-104)T polymorphism was a risk factor for DR in T2DM patients (OR=36, 95% CI=4.43-292.85, *p*=0.001), but not for C(-9)G Polymorphism (OR = 1.28, CI 95% = 0.48-3.38, *p*=0.621).

There are five haplotypes found in this study: CC/CC, CC/CG, CC/GG, CT/CC and CT/CG. As shown in Table **3**, haplotype CC/CC was a protective factor for DR (OR= 0.198; *p*=0.002), while haplotypes CT/CC and CT/CG were risk factors for DR (OR=15.58, *p*=0.002 and OR=2.29, *p*=0.005 respectively).

## 4 DISCUSSION

In this study, we found two new polymorphisms, the C-(-104)T dan C(-9)G at the regulatory region of ALR2 gene that has not been reported previously. Previous studies conducted in Australia, Chinese, Japanese, and Indonesian population found a C(-106)T polymorphism, which was reported to be correlated with DR [10, 15-17]. The C(-106)T polymorphism was also found in Finland with 85 and 126 nondiabetic patients and Brazil with 64 diabetic patients and 55 nondiabetic patients [18, 19]. In this study, we found a different polymorphic position (C(-104)T) compared to other studies (C(-106)T), in particular, with another study in Indonesian population, which included a similar number of samples (40 DR and 40 non-DR) [17]. The difference might be caused by different methods used to detect polymorphism. The study conducted by Cahyono *et al*., (2011) as well as other studies except for Li *et al*., (2001), who used PCR-SSCP and sequencing, found the T nucleotide at position -106 by employing PCR-RFLP for their detection method with BfaI restriction enzyme [2, 17]. This restriction enzyme recognized a C and a T at CTAG restriction site. In our study, C(-104)T was found based on sequencing results that were confirmed by BLAST with the reference sequence in gene bank (sequence ID: gb|U72619.1|HSU72619) as reported by Ko *et al*., (1995) [14]. BLAST result indicated that C(-106)T polymorphism was shifted two bp upstream to position -104. Further analysis using multiple alignments found that the C(-104)T polymorphism in this study was identical with C(-106)T.

Another ALR2 gene polymorphism, the C(-12)G also located at the regulatory region, was reported to be associated with DR in 123 patients with DR and 145 without DR in Chinese population [2]. BLAST result indicated that C(-9)G was shifted three bp upstream to position -9. As with the other polymorphism, further analysis using multiple alignments based on the location of -106 showed that the C(-9)G that was observed in this study was precisely the same as the C(-11)G.

Glycemic control, blood pressure, and duration of DM are important risk factors for DR. Thomas *et al*., (2012) reported that DR has strong association with the duration of diabetes and inadequate glycaemic control in Caucasian, African-Indigenous, Asian and mixed ethnics [20]. A total of 1,537 persons with type 1 and 3,978 with type 2 diabetes were included. It was reported that hypertension is strongly associated to DR in T1DM, but not in T2DM. On the other hand, in a cohort association study that evaluated 9 gene candidates associated with DR in 345 T2DM patients with DR and 365 without DR in South of India, it was reported that blood glucose, HbA1c, blood pressure and duration of diabetes were not significantly different between T2DM with and without DR [21]. In addition, they reported that duration of DM or poor glycemic control was not associated with DR. Conflicting results reported by different studies with various genetic background reflect the complexity of risk factors associated to DR. In our research, we could not exclude the possible role of significantly higher systolic blood pressure in the case group as one risk factor for DR in addition to the polymorphism at the regulatory region of ALR2 gene. Notably, the glycaemic control as indicated by HbA1c and duration of DM were comparable in both the groups (Table **1**).

In our study, we observed that C(-104)T in the regulatory region of ALR2 gene is a risk factor for DR in T2DM patients in Bali. The promoter region of ALR2 gene contains TATA box and CCAAT promoter elements showed significant transcription activities, located between +31 and -609 bases (640 bp). Cloning of this 640 bp fragment showed that deletion of -609 until -186 bases did not affect transcriptional activities of ALR2 gene [22].The promoter region of ALR2 gene is highly polymorphic. Polymorphisms that had been studied in DR were C(-106)T and C(-12)G in the promoter region, outside the coding region for protein. This finding suggested that these polymorphisms did not affect the function of ALR2 gene. Basal activities of the promoter region of *ALR2* gene are located at a position between -192 and +31 bases. The C(-106)T and C(-12)G polymorphisms are situated at the basal region of the promoter adjacent to the CCAAT promoter element. Different alleles could affect the function of CCAAT element by changing the binding activity of nuclear factor and ultimately increased the basal transcription of *ALR2* gene [23]. In the human retinal epithelial cells with the C-106 allele at ALR2 gene, higher transcriptional activities were observed than those without the T-106 alleles [3, 24]. Since both polymorphisms of C(-104)T or C(-9)G were identical as C(-106)T and C(-12)G, these polymorphisms might also increase the transcriptional activity of ALR2 gene. Up-regulation of ALR2 expression will, in turn, increase the activity of aldose reductase enzyme, which catalyzes the conversion of D-glucose into sorbitol. Accumulation of sorbitol inside ocular lens causes osmotic stress leading to ion imbalance which triggers retinopathy [5].

Our study identified five haplotypes, CC/CC, CC/CG, CC/GG, CT/CC and CT/CG, among which CC/CC haplotype was a protective factor (OR=0.198, *p*=0.002) for DR. Patients with T2DM in our study who had this haplotype were protected against DR despite suffering from diabetes for years. The average duration of DM from the two groups showed no significant difference (*p*=0.893), but the exact duration of DM was difficult to determine since most patients came for a check-up long after the symptoms appeared. The average duration of DM in patients who had CC/CC haplotype was 5.6 years. We further found that CT/CC (OR=15.58, *p*=0.002) and CT/CG (OR=2.29, *p*=0.005) haplotypes were risk factors for DR. CG genotype alone was not a risk factor for DR but together with the CT genotype in one locus, the haplotype CT/CG became a risk factor for DR. Additionally, in this study, we found CT/CC haplotype in the group of patients with younger age (49.75 years in average). This finding suggests that DM patients who had CT/CC alleles were more vulnerable and thus would experience early RD (early onset).

In the present study, we used a smaller number of samples compared to other studies. This number of samples was determined based on the low prevalence rate of polymorphism in our previous pilot study. We acknowledge that the limitation of our study was a small number of samples that may affect the result.

## CONCLUSION

In this study, we found two polymorphisms C(-104)T and C(-9)G, among which C(-104)T was a risk factor for DR in T2DM patients in Bali, Indonesia while C(-9)G was not. Five haplotypes were found, *i.e*. CT/CC, CT/CG, CT/GG, CC/CC and CC/CG. Haplotypes CT/CC and CT/CG were risk factors for DR, while CT/GG and CC/CG were not. Haplotype CC/CC was a protective factor for the occurrence of DR in T2DM patients in Bali, Indonesia.

## Figures and Tables

**Fig. (1):**
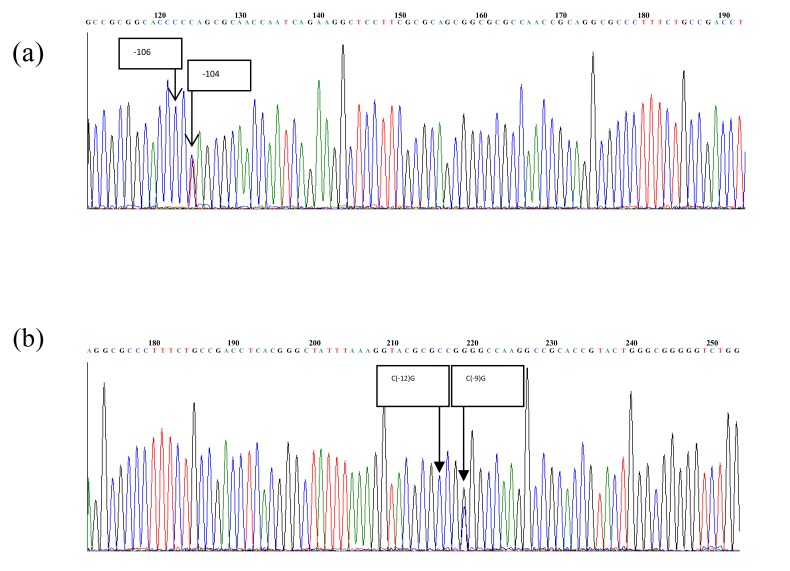
Electropherogram showing nucleotide positions of C(-104)T and C(-9)G. (a) Electropherogram of C(-106)T. Arrowindicates CT genotype at nucleotide position -104. (b) Electropherogram of C(-12)G. Arrow indicates a CG genotype at nucleotide position -9.

**Table 1:** Characteristics of subjects from case-control study.

Parameter	DR N = 35	Non-DR N = 35	*p*
Sex	–	–	–
Male (n)	16	21	0.231
Female (n)	19	14	–
Family History of DM	–	–	–
Yes (n)	21	25	0.314
No (n)	14	10	–
Age (years)	50.60±7.33	52.86±8.47	0.237
Duration of DM (years)	5.70±3.56	5.79±3.87	0.995
Systolic BP (mmHg)	132.43±13.69	125.14±11.91	**0.021**
Diastolic BP (mmHg)	84.57±7.41	81.71±7.17	0.096
Waist Circumference (cm)	89.83±10.32	89.12±10.06	0.772
BMI (kg/m^2^)	24.50±3.82	24.56±3.99	0.986
FBG (mg/dL)	183.17±69.65	146.23±48.81	**0.038**
2hppBG (mg/dL)	267.89±103.34	217.94±93.98	**0.040**
HBA1c	9.04± 2.33	8.45±2.62	0.147

The data are presented as mean ± SD; significant *p-* values are written in bold

**Table 2:** Polymorphisms distribution of ALR2 gene.

SNPs	Genotype	DR	Non-DR
C(-104)T	CC	17 (48.57)	34 (97.14)
–	CT	18 (51.43)	1 (2.86)
C(-9)G	CC	19 (54.29)	22 (62.86)
–	CG	14 (40.00)	12 (34.29)
–	GG	2 (5.71)	1 (2.86)

The data are presented as n (%)

**Table 3:** Risk factors for diabetic retinopathy.

Variable	Group		OR	95% CI		*p*
	DR (n=35)	Non-DR (n=35)		Lower	Upper	
C(-104)T	18 (51.43)	1 (2.86)	36	4.43	292.85	**0.001**
C(-9)G	14 (40.00)	12 (34.28)	1.28	0.48	3.38	0.621
Haplotype	–	–	–	–	–	–
CC/CC	8 (22.85)	21 (60.00)	0.19	0.07	0.56	**0.002**
CC/CG	6 (17.14)	12 (34.28)	0.39	0.13	1.28	0.101
CC/GG	2 (5.71)	1 (2.86)	2.06	0.18	23.83	0.555
CT/CC	11 (31.40)	1 (2.86)	15.58	1.88	128.89	**0.002**
CT/CG	8 (22.85)	0	2.29	1.73	3.05	**0.005**

Statistic analysis using Chi-square test with 95% confidence interval (*p*<0.05); significant *p* values are written in bold
